# Supplementary material for: MicroRNA-203 represses selection and expansion of oncogenic Hras transformed tumor initiating cells
Source: eLife. 2015 Jul 23;4:e07004. doi: 10.7554/eLife.07004 (PMC4536367; doi:10.7554/eLife.07004)
Supplement: Supplementary file 1. — Sequencing mapping statistics. DOI: http://dx.doi.org/10.7554/eLife.07004.023 [file elife07004s004.docx]

Small RNA-Seq Mapping Statistics

Vector HRASG12V Total Epidermis P4

|  | rep. 1 | rep. 2 |  | rep. 1 | rep. 2 |  | miR-203+/+ | miR-203-/- |  |
| --- | --- | --- | --- | --- | --- | --- | --- | --- | --- |
| Raw Reads | 772,307 | 5, 440, 980 |  | 7,356,089 | 7,846,395 |  | 2,789,021 | 3,968,317 |  |
| Reads Remaining after Trimming | 713,808 | 5,047,466 |  | 6,920,037 | 7,074,027 |  | 2,066,472 | 3,233,833 |  |
| Reads Aligned to miRNA | 73,169 | 508, 760 |  | 1,007,172 | 731,030 |  | 1,210,218 | 1,813,062 |  |

3Seq Mapping Statistics

miR-203+/+ Vector miR-203-/- Vector miR-203+/+ HRASG12V miR-203-/- HRASG12V

|  | rep. 1 | rep. 2 | rep. 1 | rep. 2 | rep. 1 | rep. 2 | rep. 1 | rep. 2 |
| --- | --- | --- | --- | --- | --- | --- | --- | --- |
| Raw Reads | 21,353,639 | 21,014,713 | 20,805,674 | 20,253,567 | 18,460,461 | 17,041,451 | 18,264,934 | 17,133,047 |
| Reads Uniquely Mapped | 17,914,434 | 17,447,436 | 18,348,508 | 16,608,243 | 15,832,338 | 14,320,434 | 14,802,086 | 14,464,448 |
| Unique Alignment (%) | 83.89% | 83.80% | 88.19% | 82.00% | 85.76% | 84.03% | 81.04% | 84.42% |

Ribo-Seq Mapping Statistics

miR-203+/+ miR-203-/-

|  | rep. 1 | rep. 2 | rep. 1 | rep. 2 |  |
| --- | --- | --- | --- | --- | --- |
| Raw Reads | 17,486,318 | 22,770,301 | 14,733,775 | 20,762,514 |  |
| Reads Remaining after Trimming | 16,945,919 | 21,995,669 | 14,154,906 | 20,169,723 |  |
| Reads Mapped to rRNA | 2,615,233 | 5,357,438 | 3,295,184 | 2,809,327 |  |
| Reads Mapped to tRNA | 2,453,229 | 2,586,723 | 1,150,757 | 3,646,802 |  |
| Reads Mapped to ncRNA | 1,550,534 | 2,202,004 | 1,496,549 | 2,126,097 |  |
| Reads Uniquely Mapped to CDS | 4,396,226 | 6,839,613 | 4,998,762 | 5,469,180 |  |
| Unique CDS Alignment Percentage | 25.14% | 30.04% | 33.93% | 26.34% |  |

Ago2 HITS-CLIP Mapping Statistics

Primary Keratinocytes

|  | rep. 1 | rep. 2 | rep. 3 | rep. 4 |
| --- | --- | --- | --- | --- |
| Raw Reads | 18,629,300 | 35,977,426 | 31,838,598 | 31,628,093 |
| Reads Aligned to miRNA | 930,455 | 2,788,847 | 4,279,814 | 2,282,176 |
| Non-miRNA reads without sequence duplicates | 794,971 | 1,181,332 | 825,478 | 821,509 |
| Reads uniquely aligned to genome | 317,710 | 479,380 | 287,440 | 329,308 |
| Combined Reads uniquely aligned to 3'UTRs | 228,109 |  |  |  |
| Combined 3'UTR Peaks | 6,770 |  |  |  |
| Combined 3'UTR Peaks containing miR-203 seed match | 117 |  |  |  |

**Supplemental File 1. Sequencing Mapping Statistics**
